# Anterior temporal lobe, word comprehension, and physiology of atrophy in semantic primary progressive aphasia

**DOI:** 10.1080/13554794.2025.2587123

**Published:** 2025-11-17

**Authors:** Jordan Q. Behn, Elena Barbieri, M. Marsel Mesulam, Borna Bonakdarpour

**Affiliations:** a Mesulam Institute for Cognitive Neurology and Alzheimer’s Disease, Northwestern University, Chicago, IL, USA; b Department of Physical Medicine and Rehabilitation, Northwestern University, Chicago, IL, USA; c Department of Neurology, Northwestern University, Chicago, IL, USA

**Keywords:** TDP-C, MRI, PET, language, atrophy, neuroimaging, PPA

## Abstract

Peak focal atrophy in the anterior temporal lobe (ATL) highlights the critical role of this area for word comprehension in semantic variant primary progressive aphasia (svPPA). However, the assumption that peak atrophy sites are specific markers of dysfunctional brain sites, and therefore reliable variables for clinicopathologic correlations, has not been rigorously tested. Using structural MRI and FDG-PET, we assessed atrophy and hypometabolism in 32 individuals with PPA (11 svPPA) and 10 healthy controls. Word comprehension was measured using the Peabody Picture Vocabulary Test. Voxel-based morphometry and standardized uptake value ratios were used to generate atrophy and hypometabolism maps. Two-sample t-tests compared svPPA and controls, and regression analyses evaluated the relationship between imaging metrics and word comprehension. Findings revealed significant bilateral ATL atrophy and hypometabolism (left > right). Structural and metabolic measures were independently associated with impaired comprehension. There was substantial overlap between atrophy and hypometabolism within the ATLs, with dysfunction extending into posterior temporal regions. However, there was no evidence of peak hypometabolism in traditional Wernicke’s area. Degeneration – both anatomical and metabolic – of the ATL serves as a robust predictor of comprehension impairment, highlighting its role a critical locus for word comprehension.

## Introduction

Neurodegenerative diseases often display substantial variability in anatomical patterns and progression across individuals. However, semantic variant primary progressive aphasia (svPPA), caused by frontotemporal lobar degeneration with transactive DNA-binding protein 43 type C pathology (TDP-C) stands out for its consistent early and selective involvement of the anterior temporal lobe (ATL) (Barbieri et al., 2025; M. M. Mesulam, 2023; Mesulam et al., 2023; Stocks et al., 2025). This anatomical specificity provides a unique opportunity to study the structure and function of the ATL.

The anterior temporal lobe is of particular interest as, despite it not being part of the classic language network, it has been more recently found to play a key role in object naming and word comprehension (i.e., verbal semantics) (Hurley et al., 2015; Mesulam et al., 2013, 2014). This finding, reproduced on multiple occasions in laboratories around the globe, has been based on patients with svPPA, who invariably have peak atrophy confined to the left anterior temporal lobe (Visser et al., 2010; Wang et al., 2023).

Given the deviation of this finding from classic aphasiology, it becomes particularly important to explore the assumption that areas of peak atrophy provide an adequate map of dysfunction. Postmortem studies have shown that areas of peak atrophy during life still maintain many viable neurons (Mesulam et al., 2023). Damage without atrophy can also occur. Postmortem examination of TDP-C reveals dystrophic neurites in many cortical areas that fail to be detected as atrophic by structural MRI (Kawles et al., 2023; Mesulam et al., 2023). Understanding these differences can be addressed, at least in part, by the addition of metabolic mapping. Are areas of peak atrophy shown by MRI metabolically active, as might be expected by the presence of residual neurons even at death? Are there areas of the brain without MRI-detectable significant atrophy that are nonetheless hypometabolic? It is the second question that motivated this report. Previous reports of dissociation between atrophy and hypometabolism in Alzheimer’s disease (Chételat et al., 2008) have raised mechanistic questions about non-atrophy drivers of metabolism unique to the disease. This precedent supports the use of FDG-PET as a complementary measure to MRI in the study of svPPA, where similar questions about structure-function relationships remain unresolved. In this study, using fluorodeoxyglucose positron emission tomography (FDG-PET), we aimed to assess the relationship between structural atrophy and hypometabolism in svPPA, with a specific focus on the anterior temporal lobe.

Previous studies have demonstrated that clinical presentation of svPPA, together with predominant anterior left temporal lobe atrophy, is highly associated with the TDP-43 type C pathology at autopsy (Mesulam et al., 2023; Montembeault et al., 2018). This strong association allows for clinicopathological inferences in living patients, particularly when structural and functional imaging patterns closely mirror those seen in autopsy-confirmed cases. Our study therefore uses svPPA and related temporal lobe neurodegeneration as a proxy for investigating patterns that are likely to reflect underlying TDP-C pathology. We hypothesized that in svPPA, the ATL would emerge as a major site of pathophysiology across neuroimaging modality, as evidenced by the overlap of atrophy and hypometabolism.

By further characterizing the established relationship between neurodegeneration in the ATL with svPPA, we can more closely examine the semantic impairments that characterize the syndrome. This focus is necessary as there has been controversy over the exact neuroanatomic underpinning of word comprehension when comparing PPA caused by neurodegeneration to stroke-induced aphasia (Hillis et al., 2017; Matchin et al., 2022; Mesulam et al., 2015). To address this gap and build on our prior work showing correlations between atrophy and impaired word comprehension (Mesulam et al., 2019), the present study incorporates cortical metabolic measurements to provide a complementary perspective on the relationship between atrophy, hypometabolism and word comprehension – an approach that has not previously been applied to a PPA cohort. We hypothesized that regardless of PPA subtype, greater impairment in word comprehension would correlate with greater atrophy and hypometabolism within the ATL. Examining these questions in parallel underscores the central role of the left ATL, linking both structural and metabolic alterations directly to observed word comprehension deficits.

## Materials and methods

This study was approved by the Northwestern University Institutional Review Board (IRB), and all participants provided informed consent in accordance with IRB guidelines.

### Participants

Thirty-two right-handed participants with PPA (mean age 67.0 ± 6.3; 17 female) and 10 healthy controls (mean age 65.8 ± 5.7; 6 female), who had both structural MRI and FDG-PET scans collected at a single visit, were included in this study (Table 1). Participants were recruited from a larger cohort of individuals with PPA enrolled in the Northwestern University PPA Program at the Mesulam Institute. Clinical diagnosis of PPA was made by author M.M. M., in accordance with Gorno-Tempini et al. (2011) based on criteria of a progressive language decline caused by neurodegenerative disease that was not accompanied by significant impairment in other cognitive domains. A subgroup was created of the 11 PPA participants diagnosed clinically with semantic variant PPA. SvPPA was diagnosed based on evidence of impaired confrontation naming and single-word comprehension arising in the context of spared repetition, grammatical competence and speech production (to differentiate svPPA from other PPA variants) and preserved object knowledge (to differentiate svPPA from semantic dementia), in accordance with previously published criteria (Mesulam et al., 2012). The remaining PPA cohort consisted of participants with agrammatic (*n* = 9), logopenic (*n* = 9), and mixed agrammatic/logopenic variant (*n* = 3) PPA.

Enrolled PPA participants were evaluated using a language assessment battery that is described in more detail in previous studies (Mesulam et al., 2012). All enrolled participants had only mild aphasia as characterized by a Western Aphasia Battery Aphasia Quotient score of greater than 80. Single-word comprehension was tested using the Peabody Picture Vocabulary Test (PPVT), a test for word comprehension that asks the participant to point to pictures of words spoken by the administrator. The PPVT does not require any speaking, reading, or writing by the participant (Dunn & Dunn, 2007). In this study, all participants were administered the same full set of items, rather than using the adaptive stopping rules of the original design, to allow direct comparison of scores across individuals. This procedure follows established administration protocols used at our center that have been previously published and discussed (Mesulam et al., 2009, 2012).

### Neuroimaging

#### Structural magnetic resonance imaging (MRI) acquisition and preprocessing

Structural T1-weighted MRI scans were acquired using MPRAGE sequences on a 3 Tesla Prisma scanner (slice thickness = 1 mm; TR = 2300 ms; TE = 2.91 ms; flip angle = 9°; FOV = 256 × 256 mm). Following manual reorientation to the AC-PC line, T1 images were preprocessed using the CAT12 toolbox (Gaser et al., 2024) within SPM12 (Friston et al., 1994).

Pre-processing in CAT12 takes place in two steps: an initial pre-processing that includes denoising, bias correction, and affine registration using SPM unified segmentation (Ashburner & Friston, 2005), and a refined voxel-based pre-processing including – among others – skull stripping, local intensity transformation of all tissue classes, adaptive maximum a posteriori (AMAP) segmentation, co-registration, and normalization to a common reference space using Geodesic Shooting (Ashburner & Friston, 2011). This process also includes calculation of total intracranial volume as a sum of gray matter, white matter and cerebrospinal fluid volume. Modulated, normalized (1.5 × 1.5 × 1.5 mm^3^) GM volume images were ultimately smoothed using an 8 mm kernel. For a more detailed description of the pre-processing steps, the reader is referred to Lukic et al. (2021) and the CAT12 Manual (https://neuro-jena.github.io/cat12-help/). GM volume images were checked for quality in CAT12, which computes an image quality rating (IQR), i.e., a weighted measure that combines information about noise contrast ratio, inhomogeneity contrast ratio, and resolution (Barbieri et al., 2024).

#### Fluorodeoxyglucose positron emission tomography (FDG-PET) acquisition and preprocessing

FDG-PET scans were acquired on a Biograph40 scanner, following ADNI-3 PET protocols (30 minutes of imaging in six 5-minute sessions, acquired 30–60 minutes post-injection). All images were manually reoriented to the AC-PC line, then preprocessed using SPM12 running in MATLAB 2022b.

Individual subjects’ standardized uptake value (SUV) maps were averaged across the 6 sessions, then coregistered and normalized to subject-specific T1 images. Partial volume correction was applied using the PETPVE12 toolbox (Gonzalez-Escamilla et al., 2017). After correction, SUV ratio (SUVR) maps were calculated voxel-wise using each individual’s whole-brain average metabolism as reference. Whole-brain scaling was chosen as this analysis was designed to emphasize relative spatial patterns rather than absolute global values. SUVR maps were spatially normalized to the CAT12 MNI template and smoothed with a 6 mm FWHM Gaussian kernel to account for intersubject anatomical differences.

## Statistical analysis

### Comparison of PPA groups to healthy control group

Statistical analysis of neuroimaging data was performed using SPM12 running in MATLAB 2022b. Normalized gray matter and SUVR maps of the svPPA group (*n* = 11) were compared to those of healthy controls (*n* = 10) via a two-sample t-test to create group-level maps of atrophy and hypometabolism, controlling for total intracranial volume. Imaging contrasts were restricted to svPPA because our hypothesis was concerned with ATL involvement and TDP-43 type C pathology and including non-svPPA subtypes (with distinct atrophy/hypometabolism patterns) would dilute subtype-specific effects. Resultant atrophy and hypometabolism T-maps were thresholded at a cluster-defining threshold of *p* < 0.001, and cluster-level FWE corrected at *p* < 0.05. A quantitative measure of similarity between atrophy and hypometabolism was assessed by calculating the Dice similarity coefficient (Dice, 1945).

### Neuroimaging measure correlation with single-word comprehension

A multiple regression model was implemented via SPM’s batch processing to analyze the relationship between hypometabolism and atrophy with single-word comprehension. Normalized and smoothed metabolism and gray matter maps for each of the 32-person all-PPA group were used as inputs, with PPVT score used as the predictor variable while controlling for participant sex, age, total intracranial volume, and disease duration. All PPA participants were included in the behavioral multiple regression analysis to maximize variance (both in behavior and atrophy patterns) and power. Additionally, this analysis was designed to address the neuroanatomic basis of impaired word comprehension independent of underlying pathology. PPA subjects scored an average of 86% ± 14% correct on the PPVT. Maps were thresholded at a cluster-defining threshold of *p* < 0.001, and cluster-level FWE corrected at *p* < 0.05.

## Results

### Atrophy and hypometabolism maps (two-sample t-test)

**Figure 1.**
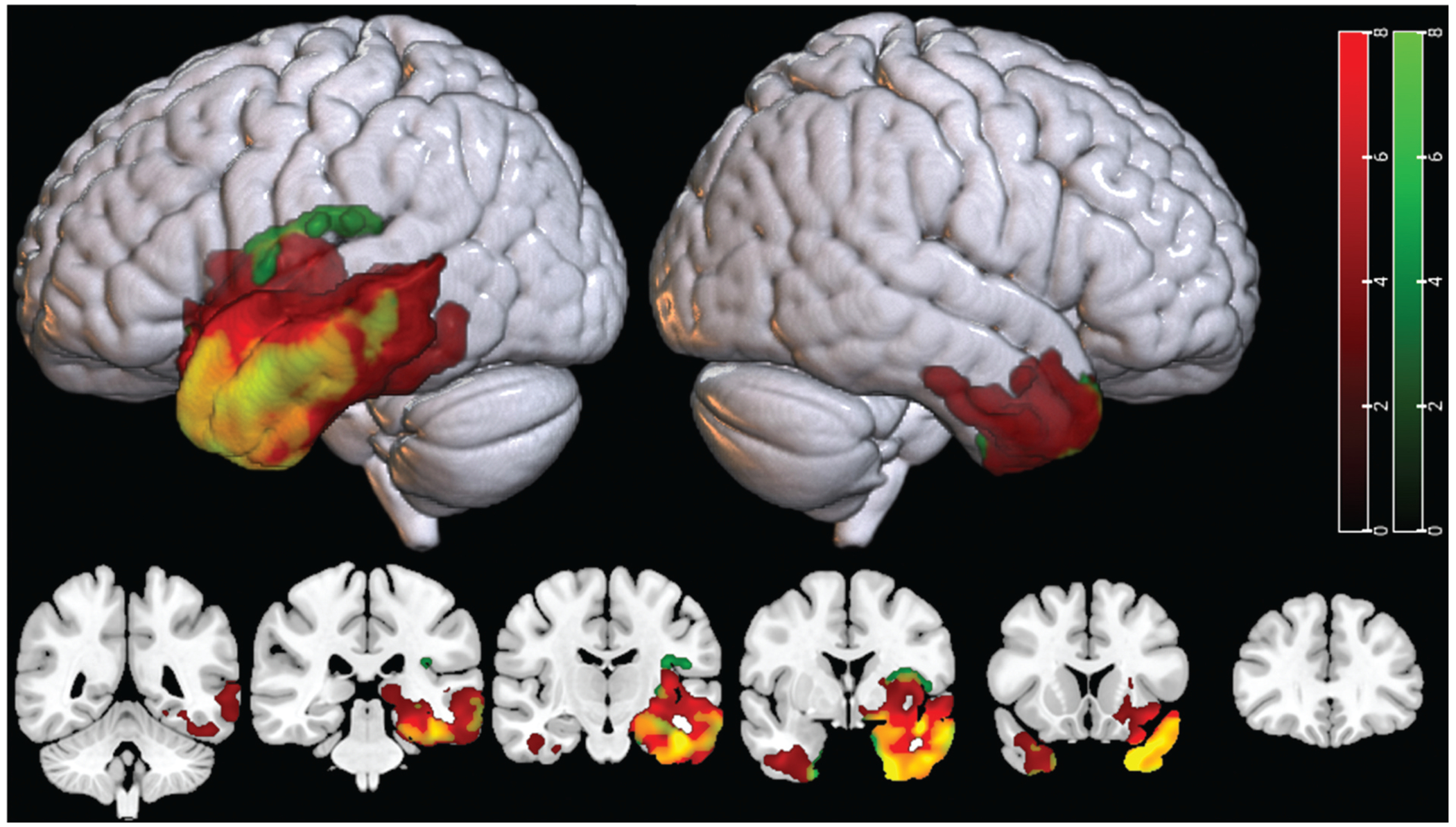
Atrophy (red) and hypometabolism (green) thresholded t-maps for people with semantic PPA (*n* = 11). colored regions indicate significantly (*p* < 0.001, FWEc *p* < 0.05) decreased gray matter (red) or SUVR signal (green) as compared to healthy participants (*n* = 10). Yellow indicates areas with both significant hypometabolism and atrophy.

The results of the two-sample t-tests revealed areas of significant decreases (*p* < 0.001, FWEc *p* < 0.05) in both gray matter volume (red clusters) and brain metabolism (green clusters) in the svPPA group compared to the controls (Figure 1). The svPPA group showed the greatest decreases in both gray matter and glucose metabolism in the anterior temporal lobe (overlap shown in yellow) as compared to controls. More modest decreases in both measures extended to the middle and posterior temporal lobe, with atrophy extending slightly beyond the boundaries of hypometabolism in these areas. Additional decreases were observed in insular and parahippocampal regions, as well as in homologous right hemisphere ATL regions, albeit to a lesser degree. FDG-PET measures of hypometabolism extended beyond the areas of atrophy in a total of 23,361 voxels, exclusively in the left hemisphere and most substantially in the posterior insula. Atrophy extended beyond hypometabolism in 16,150 voxels, primarily in posterior temporal areas. The intersection of the two measures had an extent of 28,103 voxels with a Dice similarity coefficient between the two regions of 0.59.

### Correlation with single-word comprehension

**Figure 2.**
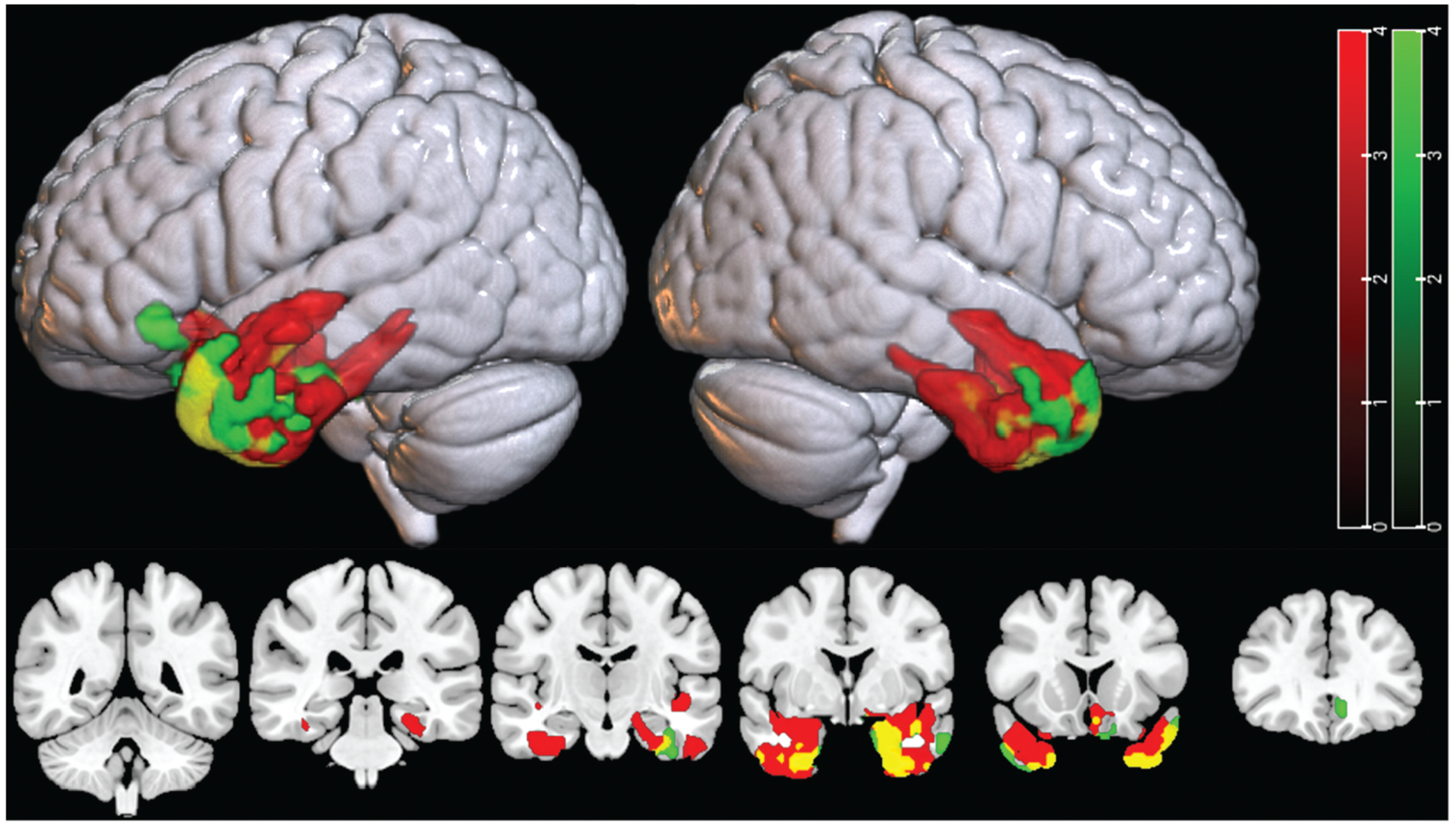
Thresholded correlation maps between PPVT single-word comprehension scores with atrophy (red), hypometabolism (green), or both (yellow). Colored regions indicate regions that significantly (*p* < 0.001, FWEc *p* < 0.05) correlate with decreased gray matter (red) or SUVR signal (green). Areas that correlate with both neuroimaging measures are yellow.

Figure 2 shows the results of the regression analysis using the complete cohort of participants with PPA (*n* = 32). It highlights regions where neuroimaging measures are significantly and positively correlated with accuracy on our selected measure of single-word comprehension (PPVT). Red clusters indicate areas where lower PPVT scores significantly correlated with decreased gray matter volume (hence, with greater atrophy), green clusters with decreased metabolism (hence with greater hypometabolism), and yellow regions represent areas of overlap between the two modalities. Both imaging modalities show peak correlation in the left temporal pole, with significant associations also observed in the anterior insula, parahippocampal regions, and frontal regions (Table 2).

## Discussion

In this study, we explored whether the relationship of structural atrophy with metabolic mapping could further clarify the physiology of atrophy and the legitimacy of clinicoanatomical correlations based on the distribution of peak atrophy sites. This multimodal approach is novel in that it combines whole-cohort regression analyses of word comprehension with svPPA-specific imaging contrasts, thereby showing that both structural and metabolic markers in the ATL independently predict language deficits while also reaffirming the unique vulnerability of this region in svPPA. Our findings show that atrophy and hypometabolism largely overlap in the left ATL in patients with svPPA which is most commonly caused by TDP-C. The moderate Dice similarity coefficient of 0.59 reflects a significant overlap given the differences in resolution and sensitivity of the two neuroimaging measures (Dice, 1945; Grothe & Teipel, 2016; Taha & Hanbury, 2015). This overall co-occurrence may reflect that, in svPPA, decreases in metabolism and gray matter volume occur mainly in tandem, particularly in regions of peak vulnerability such as the ATL.

While the observed overlap indicates that both measures capture damage to the ATL, smaller regions showing only hypometabolism or only atrophy reveal that structural and functional changes may be partially dissociable rather than strictly overlapping. For example, we found an area of hypometabolism within the left anterior insula in the absence of atrophy. While neurodegeneration in svPPA starts in the ATL, left posterior insular degeneration may occur later in the course of the disease (Seeley, 2010). Similarly, areas of exclusive atrophy are seen posterior to those of overlap within the temporal lobe, again reflecting areas that are known to undergo neurodegeneration in later stages of the disease. These differences may reflect the distinct cytoarchitecture and connectivity of each region, leading to partially divergent structural and functional trajectories as the disease progresses.

In line with results in previous studies that have looked at semantic dementia (Desgranges et al., 2007; Diehl et al., 2004), our results show that in svPPA with suspected FTLD TDP-C, hypometabolism appears to be closely linked to structural atrophy. This is not the case in Alzheimer’s disease (which primarily causes the logopenic variant of PPA), as hypometabolism often precedes and exceeds atrophy in several brain regions, including the posterior cingulate, angular gyrus, and parahippocampal cortex, suggesting that the underlying pathology affects neuronal metabolism before atrophy (Chételat et al., 2008; Rodriguez-Oroz et al., 2015). A recent study in Alzheimer’s disease (Strom et al., 2022) found that hypometabolism is primarily driven by local atrophy and tau pathology, suggesting that tau-related neuronal dysfunction may explain the dissociation between metabolism and atrophy that is not seen in FTLD TDP-C. These disease-specific relationships between structural and metabolic change are necessary for interpreting their clinical consequences. In svPPA, the significant overlap of atrophy and hypometabolism in the ATL provides a direct link to the observed word comprehension deficits.

There has been ongoing debate about the locus of word comprehension since 2015. Stroke studies suggest that the posterior superior temporal gyrus, traditionally called Wernicke’s area, is primarily responsible (Hillis et al., 2017; Matchin et al., 2022). In contrast, findings from PPA research suggest damage to the left ATL as the underlying mechanism for anomia and impaired word comprehension (Mesulam et al., 2015, 2019). By examining word comprehension across the full PPA cohort, with the introduction of FDG-PET correlations as a novel measure alongside established atrophy analyses, this study highlights the ATL as the critical region responsible for impaired word comprehension in PPA.

TDP-C inclusions take the form of long, thick dystrophic neurites, predominantly in the superficial cortical layers. These neurites are believed to represent dystrophic apical dendrites of layer III and V pyramidal neurons, which play crucial roles in complex cortical computations (Kawles et al., 2022; Mesulam et al., 2023). The resultant neurocomputational disruption at the ATL, which is located at the downstream confluence of converging information processing streams and appears responsible for the impaired word comprehension. This interpretation is reinforced by the results of the present study, as correlates of impaired word comprehension across both structural and metabolic measures were centered in the ATL, not within the boundaries of the classical Wernicke’s area.

This study has several limitations. First, we only looked at PPA patients with both MRI and FDG-PET scans. This cohort was relatively small, which may limit statistical power and the generalizability of the findings. Second, autopsy confirmation of TDP-C was not available, so underlying pathology is inferred from clinical presentation and imaging patterns. Third, the cross-sectional design prevents temporal sequence of hypometabolism and atrophy, making it difficult to determine causality. Finally, differences in spatial resolution and sensitivity between MRI and FDG-PET may reduce the precision of comparisons between structural and metabolic measures. Despite these limitations, the study provides valuable insight into the convergence and divergence of structural and functional changes in the ATL and their relationship to word comprehension deficits.

In summary, we found significant overlap between atrophic and hypometabolic regions of the ATL in individuals with svPPA and showed that ATL hypometabolism correlates with word comprehension impairment. These findings enhance our understanding of well-established atrophy-behavior correlations by showing that atrophy and metabolism are tightly linked in this disease, refining our understanding of the pathophysiology of svPPA and its associated symptoms. Future research leveraging integrative imaging techniques may deepen our comprehension of disease mechanisms and progression and open pathways to earlier diagnosis and treatment strategies.

## Figures and Tables

**Table 1.** Participant demographic breakdown of control, all PPA, and svPPA groups.

Group	Number of Participants (n)	Mean Age (years)	Sex (M/F)
Control	10	65.8 ± 5.7	4 / 6
All PPA	32	67.0 ± 6.3	15 / 17
svPPA	11	64.5 ± 6.7	6 / 5

**Table 2.** Coordinates for clusters with significant correlation (*p* < 0.001, FWEc *p* < 0.05) between word comprehension score (PPVT) and cortical volume/metabolism.

	Peak T-stat	Peak region	Peak MNI Coordinates (mm)
Gray Matter Volume	6.21	Left temporal pole	−22, 14, −45
	8.54	Right temporal pole	32, −4, −28
SUVR	6.70	Left temporal pole	−38, 22, −42
	6.04	Right temporal pole	20, 10, −42

## Data Availability

Data and code are available upon official request using the following e-mail: mesulam-center@northwestern.edu
